# Thermal management in annular fin using ternary nanomaterials influenced by magneto-radiative phenomenon and natural convection

**DOI:** 10.1038/s41598-023-36418-4

**Published:** 2023-06-12

**Authors:** Khalid Abdulkhaliq M. Alharbi, Adnan, Mutasem Z. Bani-Fwaz, Sayed M. Eldin, Ali Akgul

**Affiliations:** 1 Mechanical Engineering Department, College of Engineering, Umm Al-Qura University, 24382 Makkah, Saudi Arabia; 2 Department of Mathematics, Mohi-ud-Din Islamic University, Nerian Sharif, AJ&K 12080 Pakistan; 3 Department of Chemistry, College of Science, King Khalid University, P. O. Box 9004, 61413 Abha, Saudi Arabia; 4 Center of Research, Faculty of Engineering, Future University in Egypt, New Cairo, 11835 Egypt; 5 Department of Computer Science and Mathematics, Lebanese American University, Beirut, Lebanon; 6 Department of Mathematics, Art and Science Faculty, Siirt University, 56100 Siirt, Turkey; 7 Mathematics Research Center, Department of Mathematics, Near East University, Near East Boulevard, 99138 Nicosia/Mersin 10, Turkey

**Keywords:** Engineering, Mathematics and computing

## Abstract

Annular fin is a particular mechanical setup for heat transfer that varies radially and frequently utilize in applied thermal engineering. Addition of annular fin to working apparatus enhance the surface area in contact with surrounding fluid. Other potential areas of fin installation are radiators, power plant heat exchangers and also it plays significant role in sustainable energy technologies. The major objective of this research is to introduce an efficient annular fin energy model influenced by thermal radiation, magnetic forces, coefficient of thermal conductivity, heating source with addition of modified Tiwari–Das model. Then, numerical treatment performed to acquire the desired efficiency. From the results, it is scrutinized that the fin efficiency significantly improved by strengthening the physical strength of ${\alpha }_{1}, {\alpha }_{2}$ and ${\gamma }_{1}$ and the use of ternary nanofluid make it more efficient. Addition of heating source ${Q}_{1}$ make the fin more efficient and radiative number is better to cool it. The role of ternary nanofluid observed dominant throughout the analysis and the results validated with existing data.

## Introduction

The heat transport due to natural convection is probable research in the last two decades because of their widespread applications in various engineering disciplines like food engineering, electronics, chemical, mechanical and aerodynamics. Natural convection, also referred as free convection, occurs as consequence of density differences between two fluids maintained at different temperatures. Also, concrete applications of natural convection extensively found in solar systems. Several theoretical and experimental studies on free convection in nanofluids have used different working domains and geometries in which fin is an important apparatus due to their wider applications.

**Figure 1:**
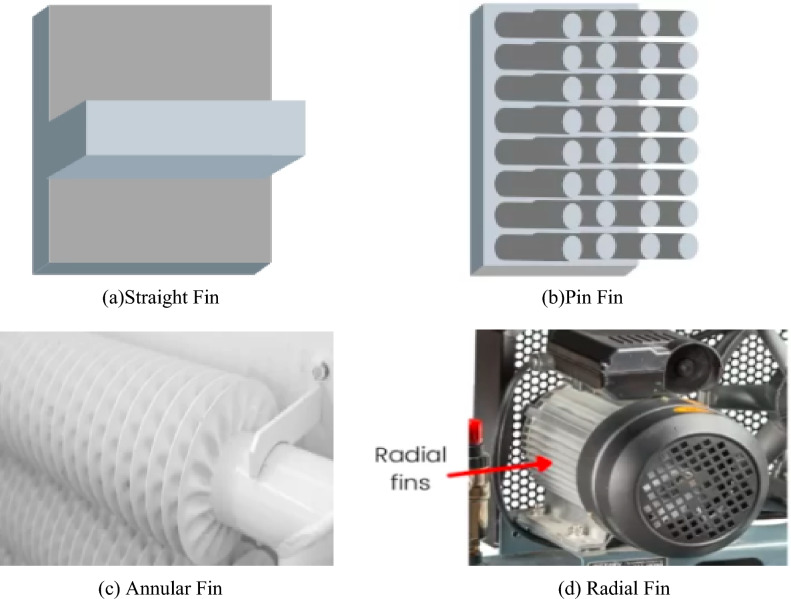
Different fin functioning geometries.

The expanded surface utilized to boost the rate of convective heat transfer is known as a fin. Fins are often employed on the surface when the heat transfer rate is insufficient to cool or heat the body without the usage of fins. As a result, the fins aid in increasing the surface exposed to the circulating media. To begin, heat is moved from the body to the fins by conduction, and then heat from the fins is transferred away via convection. The materials use for the manufacturing of fins highly depend on corrosion resistant, light weight, outstanding thermal conductivity and cast ability; thus, these are key factors in selecting the material. The fins are categorized according to their functioning condition and available area and the possible functional fins are straight fins^1–3^ radial and rectangular fins^4^, annular fin and pin fin^5,6^. These fins apparatuses further divided into subclasses based on their various cross sections. Fins of various shapes are given in Fig. 1.

In recent years, use of mono nano (see Refs^7–9^.) and bihybrid nanofluids (see Refs^10–13^.) convinced the engineers and researchers from the various applied fields to acquire their required output. In 2022, Jan et al.^14^ described the importance of hybrid nanoliquid for thermal improvement. They considered ZnO, Ni and H_2_O particles and liquid components for the analysis. Further, the effects of darcy media introduced in the study and found the velocity and temperature distributions in (ZnO-Ni)/H_2_O. Parallel to Newtonian nanoliquids, the fluids possessing the non-Newtonian behaviour are also of much significance because of their unique properties. Thus, Pattanaik et al.^15^ reported a non-Newtonian model using the concept of thermal upthrust and noticed that the presence of upthrust forces have good velocity controlling properties. Some of the diverse studies regarding the performance of nano as well as hybrid nanoliquids under additional physical aspects like time dependent thermal flux, porous media effects and homogeneous/heterogeneous chemical species and their role in the transportation of thermal gradient discussed by different researchers (see Refs^16–18^.). Recently, Ikram et al.^19^ and Pattnaika et al.^20^ made attempt to investigate the heat transmission under zero mass flux, heat source, and MHD on single phase nanoliquid, and pressure gradient diminishes over a plan plate. The studies revealed that nanoliquid are better source to acquire the required heat transmission for better industrial applications.

Kumar et al.^21^ inspired by significance of annular fin applications adjusted with multi-boiler heat transport coefficients. To achieve the remarkable efficiency of the fin, DTM scheme implemented and also validate the study with published research data of Mallick et al.^22^ and Arslanturk^23^. They observed that thermal radiations and heat sink/source are physical tools which can be used to boosts the performance of annular fin. Different fin models have their own significance and natural convective heat performance. Thus, Reddy and Mishra^24^ organized a comprehensive thermal performance of various fins. The comparison made between multiple fin models and check their efficiency via theoretical way. The authors observed that Darcy and Rayleigh control parameters are good to acquire the high efficiency by considering working nanoliquid and the particle movement in porous annulus. The 3D effects on fin efficiency with eccentricity over a cylinder subject to annular fins is examined by^25^. The study revealed that appropriate adjustment of disc and cylinder highly affect the heat transfer under natural convection phenomena.

**Table 1:** Published literature related to heat performance of various fins under varying physical aspects.

S.no	Studies performed	Fin shape	Physical aspects discussed	Use of nanofluid		
				Nano	Hybrid	Ternary
1	Ganeshkumar et al.^26^	Radial	Analysis under thermal radiations and TD thermal conductivity using DTM in the absence of nanofluid	$\times$	$\times$	$\times$
2	Rai et al.^27^	Circular	Curvature and nanoparticles concentration	$\surd$	$\times$	$\times$
3	Poursharif et al.^28^	Triangular	Porous surface and magnetic field	$\surd$	$\times$	$\times$
4	Haq et al.^29^	Parallel Fins	Natural convection and SWCNTs nanomaterial	$\surd$	$\times$	$\times$
5	Hamida et al.^30^	Different fins	Examined the effects of electric field with various voltage stages	$\times$	$\surd$	$\times$
6	Manohar et al.^31^	Spherical porous fin	Studied natural convection, radiation and internal heating source	$\times$	$\surd$	$\times$
7	Turkyilmazoglu^32^	Elastic longitudinal fins	Examined thermal efficiency influenced by stretching/shrinking and Biot number	$\times$	$\times$	$\times$
8	Jalili et al.^33^	Curved fin	Nanofluid is taken as functional fluid with convective effects	$\surd$	$\times$	$\times$
9	Naphon et al.^34^	Minin rectangular fin	Discussed the heat transmission with heat sinks	$\surd$	$\times$	$\times$
10	Current research	Annular Fin source setup	Natural convective heat transfer, coefficient of thermal conductance, thermal radiations, heating source and magnetic field	$\surd$	$\surd$	$\surd$

Recently, Ullah et al.^35^ convinced the researchers towards the study of convective fins. To enhance the fin performance, the authors integrated the effects of heating source and thermal radiations. The problem modeled for triangular type fin and then discussed the results painstakingly by examining the influence of the physical parameters.

Primarily, fins are elongating surface over the working machinery which enhance the heat transfer rate and efficiency of the object. The most common uses of fins can be found in radiators, heat exchangers, electric motors, as heat sinks in CPU and in various engineering purposes more specifically in mechanical engineering etc. All the above discussed open literature including Table 1 reveals that till date no one made attempt to examine the heat transfer performance of annular fin with rectangular shape with significant effects of natural convection, coefficient of thermal conductance, thermal radiations, internally affecting heating source and directed magnetic field using advance Tiwari–Das ternary nanoliquid model which is a probable research gap in the particular field. Thus, all these physical phenomena will address in this research via a mathematical model and observe the heat transmission performance of rectangular fin using these physical facts. Also, the analysis will be useful to predict the physical parametric ranges to make the annular fin more efficient.

Further, the audience will aware about the below significant research questions and their appropriate answers regarding the fin performance after performing the study:

- Impacts of advance Tiwari–Das model on the natural convective heat performance of annular fin?
- Among the physical effects of thermal radiations, internal heat source, magnetic field and thermal conductivity coefficient which one greatly influenced the performance of fin?
- The suitable and appropriate ranges of the parameters involved in the energy model to make the fin more functional with high efficiency.

## Formulation of the model

This section is very important because all information about the fin energy model involving various physical phenomena is discussed in depth in this section.

### Statement and annular fin structure

**Figure 2:**
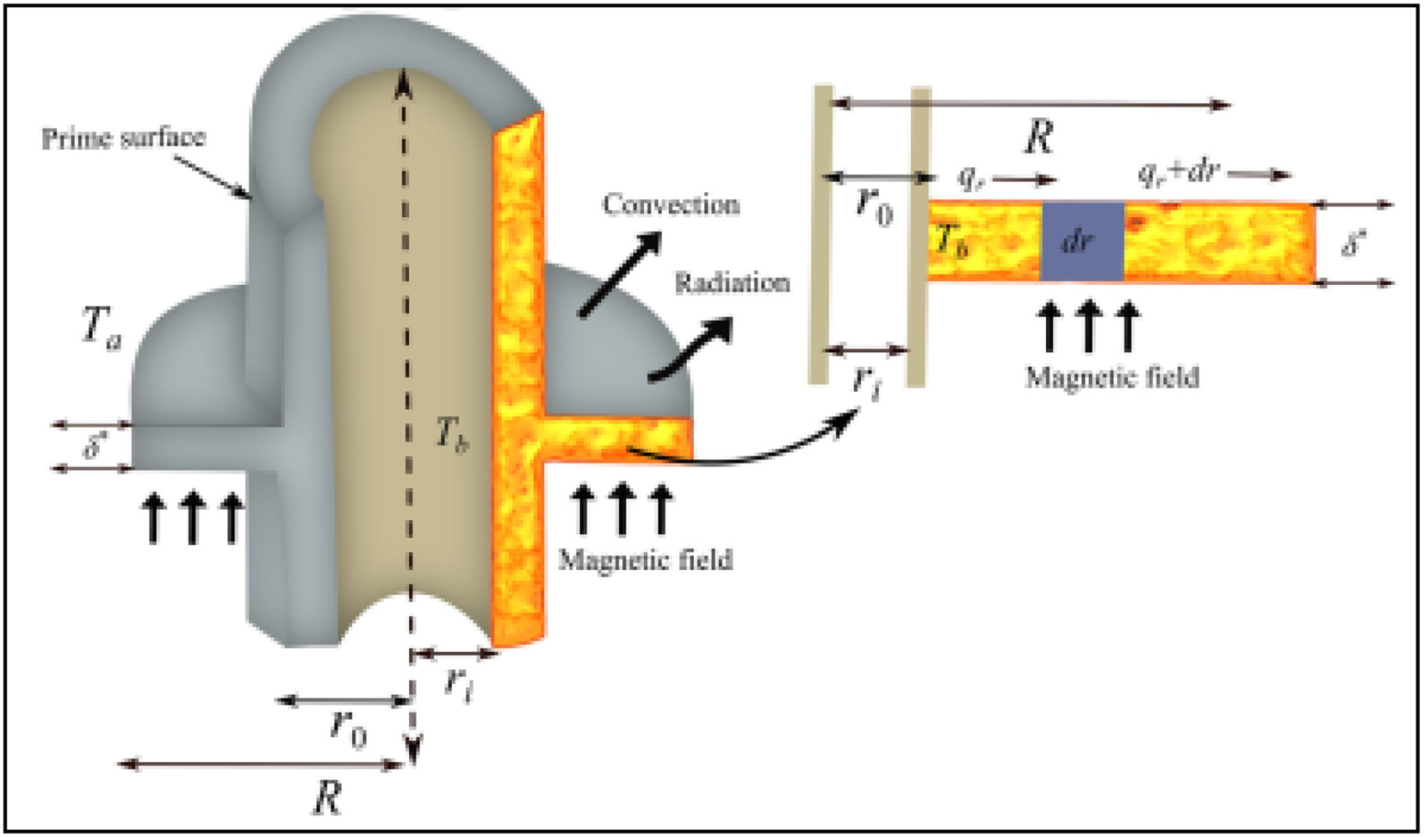
Physical appratus of annular fin with various physical possessions.

The study deals with the thermal performance of annular fin influenced by various significant physical phenomena like thermal radiation, convective-conduction, inner heat source and thermal expansion coefficient. Figure 2 illustrated the complete apparatus of annular fin in which coil is rounded about the rectangular surface. The following norms are critical in the development of energy model for annular fin:

- The fin is subject to the dimensions ${\widetilde{r}}_{0}$ (outer radius), ${\widetilde{r}}_{i}$ (internal radius), ${\widetilde{\delta }}^{*}$ (fin thickness) and ${\widetilde{A}}_{cross}$ (cross section).
- The temperature of primary surface and annular fin are maintained as ${\widetilde{T}}_{b}$ and ${\widetilde{T}}_{a}$ and one-dimensional heat dissipation is obvious as a function of $\widetilde{r}$.
- The non-transient energy model is assumed because the heat transmission in not a function of time through the fin and topmost of the fin is fine insulated.
- The energy fin model comprises the insights of magnetic field (${\widetilde{B}}_{0}$), thermal radiations and inner heat source.

According to the limitations mentioned above, the energy model of annular fin described in the succeeding way and involved physical constraints given in Table 2:

1 $$- \tilde{q}_{{\tilde{r} + d\tilde{r}}} + \tilde{q}_{{\tilde{r}}} = 2\pi \tilde{h}^{*} \tilde{r}\left( {\Lambda_{1} } \right)d\tilde{r} + \left[ {\begin{array}{*{20}c} {2\tilde{\sigma }\tilde{r}\pi \tilde{\varepsilon }\left( {\Lambda_{2} } \right)d\tilde{r}} \\ { - 2\tilde{\sigma }^{*} \tilde{r}\pi \tilde{q}^{*} \left( {\tilde{T}} \right)d\tilde{r}} \\ \end{array} } \right] + 2\pi \tilde{\delta }^{*} \left[ {\frac{{\tilde{J}_{c} \times \tilde{J}_{c} }}{{\tilde{\sigma }_{m} }}} \right]\tilde{r}d\tilde{r}$$

**Table 2:** Physical constraints involved in the energy model given in Eq. (1).

Symbol	Physical meaning	Symbol	Physical meaning
${\widetilde{h}}^{*}$	Convective heat transport coefficient	$\widetilde{q}$	Heat disperses at $\widetilde{r}$
${\widetilde{q}}^{*}(\widetilde{T})$	Heat augmentation unit/volume	$\widetilde{\epsilon }$	Fin surface emissivity
$\widetilde{\sigma }$	Stefan Boltzmann coefficient	${\widetilde{J}}_{c}$	Convective current magnitude
$\widetilde{T}-{\widetilde{T}}_{a}$	${\Lambda }_{1}$	${\widetilde{T}}^{4}-{\widetilde{T}}_{a}^{4}$	${\Lambda }_{2}$

The RHS of Eq. (1) is the physical interpretation of convective heat transfer and then the second expression represents the heat loss from the fin into neighboring region of fin. Further, heat source effects and magnetic field effects designated by third and fourth terms, respectively.

Now, when $d\tilde{r}$ approaches to zero, the following compact energy expression^27^ is obtained from Eq. (1):

2 $$- \tilde{q}^{\prime}\left( {\tilde{r}} \right) - 2\pi \tilde{h}^{*} \tilde{r}\left( {\Lambda_{1} } \right) + \left[ {\begin{array}{*{20}c} { - 2\pi \tilde{\varepsilon }\tilde{\sigma }\tilde{r}\left( {\Lambda_{2} } \right)} \\ { + 2\pi \tilde{q}^{*} \left( {\tilde{T}} \right)\tilde{\delta }^{*} \tilde{r}} \\ \end{array} } \right] - \left[ {\frac{{\tilde{J}_{c} \times \tilde{J}_{c} }}{{\tilde{\sigma }_{m} }}} \right]2\pi \tilde{\delta }^{*} \tilde{r}$$

The Fourier law of heat conduction is used to simplify the model described in Eq. (2) and then more shortened expression achieved as below:

3 $$\widetilde{q}=-\widetilde{k}\left(\widetilde{T}\right){\widetilde{A}}_{cross}\frac{d\widetilde{T}}{d\widetilde{r}} with {\widetilde{A}}_{cross}=2\pi {\widetilde{\delta }}^{*}\widetilde{r}$$

Further, the quantities comprised in in Eq. (3) described by the following physical expressions:

4 $$\left.\begin{array}{c}{\widetilde{h}}^{*}\left(\widetilde{T}\right)={\widetilde{h}}_{b}{\left[\frac{{\Lambda }_{1}}{{\widetilde{T}}_{b}-{\widetilde{T}}_{a}}\right]}^{n}\\ \widetilde{k}\left(\widetilde{T}\right)={\widetilde{k}}_{ternary}\left[1+\widetilde{k}\left({\Lambda }_{1}\right)\right]\\ {\widetilde{q}}^{*}\left(\widetilde{T}\right)={\widetilde{q}}_{a}\left[1+\widetilde{c}\left({\Lambda }_{1}\right)\right]\end{array}\right\}$$

The quantities are ${\widetilde{k}}_{ternary}$ (ternary nanofluid thermal conductivity), ${\widetilde{q}}_{a}$ (heat source), $\widetilde{c}$ (heat generation source), ${\widetilde{h}}_{b}$ (convective heat transmission coefficient) and $n$ designates the heat transmission mode. The appropriate of limits of $n$ are considered as − 6.6 and 5.0. For the many engineering applications like materials processing and nuclear reactions, these values adjust between − 3 and 3. The selection of $n$^36^ for turbulent, laminar natural convection and laminar film boiling are 0.333, 0.25 and − 0.25, respectively. The uniform heat transport, nucleate boiling and radiation correspond to the values 0, 2 and 3, respectively.

Now, use of Eq. (3) and Eq. (4) in Eq. (2) yields the following refined energy model:

5 $$\frac{d}{d\widetilde{r}}\left[2\pi {\widetilde{\delta }}^{*}\widetilde{r}{\widetilde{k}}_{ternary}\left\{1+\widetilde{k}\left({\Lambda }_{1}\right)\right\}\frac{d\widetilde{T}}{d\widetilde{r}} \right]+\left[\begin{array}{c}-2\pi \widetilde{r}{\widetilde{h}}_{b}{\left(\frac{{\Lambda }_{1}}{{\widetilde{T}}_{b}-{\widetilde{T}}_{a}}\right)}^{n}\left({\Lambda }_{1}\right)\\ -2\pi \widetilde{\epsilon }\widetilde{\sigma }\widetilde{r}\left({\Lambda }_{2}\right)\\ +2\pi {\widetilde{\delta }}^{*}\widetilde{r}{\widetilde{q}}_{a}\left(1+\widetilde{c}\left({\Lambda }_{1}\right)\right)\\ -2\pi {\widetilde{\sigma }}^{*}\widetilde{r}\left(\frac{{\widetilde{J}}_{c}\times {\widetilde{J}}_{c}}{{\widetilde{\sigma }}_{m}}\right)\end{array}\right]$$

Using $\left(\frac{{\widetilde{J}}_{c}\times {\widetilde{J}}_{c}}{{\widetilde{\sigma }}_{m}}\right)={\widetilde{\sigma }}_{ternary}{{\widetilde{B}}^{2}}_{0}{\widetilde{u}}^{2}$ in Eq. (5), the version attained with more clarification:

6 $$\frac{d}{d\widetilde{r}}\left[2\pi {\widetilde{\delta }}^{*}\widetilde{r}{\widetilde{k}}_{ternary}\left\{1+\widetilde{k}\left({\Lambda }_{1}\right)\right\}\frac{d\widetilde{T}}{d\widetilde{r}}\right]+\left[\begin{array}{c}-2\pi \widetilde{r}{\widetilde{h}}_{b}{\left(\frac{{\Lambda }_{1}}{{\widetilde{T}}_{b}-{\widetilde{T}}_{a}}\right)}^{n}\left({\Lambda }_{1}\right)\\ -2\pi \widetilde{\epsilon }\widetilde{\sigma }\widetilde{r}\left({\Lambda }_{2}\right)\\ +2\pi {\widetilde{\delta }}^{*}\widetilde{r}{\widetilde{q}}_{a}\left(1+\widetilde{c}\left({\Lambda }_{1}\right)\right)\\ -2\pi {\widetilde{\sigma }}^{*}\widetilde{r}\left({\widetilde{\sigma }}_{ternary}{{\widetilde{B}}^{2}}_{0}{\widetilde{u}}^{2}\right)\end{array}\right]$$

In the present work, it is essential to keep the fin top as insulated and then the BCs achieved as follows:

7 $$\left.\begin{array}{c}At \widetilde{r}={\widetilde{r}}_{i}: \widetilde{T}={\widetilde{T}}_{b}\\ At \widetilde{r}={\widetilde{r}}_{0}: \frac{d\widetilde{T}}{d\widetilde{r}}=0\end{array}\right\}$$

For further simplification of the model, the appropriate variables defined below:

8 $$\left.\begin{array}{c}\beta =\frac{\widetilde{T}}{{\widetilde{T}}_{b}}, {\alpha }_{2}=\frac{{\widetilde{T}}_{a}}{{\widetilde{T}}_{b}},{\alpha }_{1}=\widetilde{k}{\widetilde{T}}_{b},{\gamma }_{1}=\widetilde{c}{\widetilde{T}}_{b},\eta =\frac{\widetilde{r}-{\widetilde{r}}_{i}}{{\widetilde{r}}_{i}},\widetilde{R}=\frac{{\widetilde{r}}_{0}}{{\widetilde{r}}_{i}}\\ {N}_{c1}=\frac{{\widetilde{h}}_{b}{\widetilde{r}}_{i}^{2}}{{\widetilde{k}}_{f}{\widetilde{\delta }}^{*}},{R}_{d}=\frac{\widetilde{\epsilon }\widetilde{\sigma }{\widetilde{r}}_{i}^{2}{\widetilde{T}}_{b}^{3}}{{\widetilde{k}}_{f}{\widetilde{\delta }}^{*}}, {Q}_{1}=\frac{{\widetilde{q}}_{0}{\widetilde{r}}_{i}^{2}}{{\widetilde{k}}_{f}{\widetilde{T}}_{b}},{M}_{1}=\frac{{\widetilde{B}}_{0}^{2}{\widetilde{u}}^{2}{\widetilde{r}}_{i}^{2}}{{\widetilde{k}}_{f}{\widetilde{T}}_{b}}\end{array}\right\}$$

### Effective models of ternary nanofluid

Additional thermal and electrical conductivities of nanoparticles and basic fluid are very important in the heat transfer performance of functional fluid. Thus, for the current research the following models (Table 3) used to make it more efficient.

**Table 3:** Modified Tiwari–Das thermal conductivity, nanoparticles properties and electrical conductivity models.

Characteristics	Empirical correlation			
Thermal conductivity	$$\frac{{\overset{\lower0.5em\hbox{$\smash{\scriptscriptstyle\smile}$}}{k}_{{\left( {Al_{2} O_{3} - CuO - Cu} \right)w}} }}{{\overset{\lower0.5em\hbox{$\smash{\scriptscriptstyle\smile}$}}{k}_{{\left( {Al_{2} O_{3} - CuO} \right)w}} }} = \frac{{\overset{\lower0.5em\hbox{$\smash{\scriptscriptstyle\smile}$}}{k}_{Cu,p3} + 2\overset{\lower0.5em\hbox{$\smash{\scriptscriptstyle\smile}$}}{k}_{{\left( {Al_{2} O_{3} - CuO} \right)w}} - 2\phi_{Cu,p3} \left( {\overset{\lower0.5em\hbox{$\smash{\scriptscriptstyle\smile}$}}{k}_{{\left( {Al_{2} O_{3} - CuO} \right)w}} - \overset{\lower0.5em\hbox{$\smash{\scriptscriptstyle\smile}$}}{k}_{Cu,p3} } \right)}}{{\overset{\lower0.5em\hbox{$\smash{\scriptscriptstyle\smile}$}}{k}_{Cu,p3} + 2\overset{\lower0.5em\hbox{$\smash{\scriptscriptstyle\smile}$}}{k}_{{\left( {Al_{2} O_{3} - CuO} \right)w}} + \phi_{Cu,p3} \left( {\overset{\lower0.5em\hbox{$\smash{\scriptscriptstyle\smile}$}}{k}_{{\left( {Al_{2} O_{3} - CuO} \right)w}} - \overset{\lower0.5em\hbox{$\smash{\scriptscriptstyle\smile}$}}{k}_{Cu,p3} } \right)}}$$ $$\frac{{k_{{\left( {Al_{2} O_{3} - CuO} \right)w}} }}{{k_{nf} }} = \frac{{\overset{\lower0.5em\hbox{$\smash{\scriptscriptstyle\smile}$}}{k}_{CuO,p2} + 2\overset{\lower0.5em\hbox{$\smash{\scriptscriptstyle\smile}$}}{k}_{nf} - 2\phi_{CuO,p2} \left( {\overset{\lower0.5em\hbox{$\smash{\scriptscriptstyle\smile}$}}{k}_{nf} - \overset{\lower0.5em\hbox{$\smash{\scriptscriptstyle\smile}$}}{k}_{CuO,p2} } \right)}}{{\overset{\lower0.5em\hbox{$\smash{\scriptscriptstyle\smile}$}}{k}_{CuO,p2} + 2\overset{\lower0.5em\hbox{$\smash{\scriptscriptstyle\smile}$}}{k}_{nf} + \phi_{CuO,p2} \left( {\overset{\lower0.5em\hbox{$\smash{\scriptscriptstyle\smile}$}}{k}_{nf} - \overset{\lower0.5em\hbox{$\smash{\scriptscriptstyle\smile}$}}{k}_{CuO,p2} } \right)}}$$ $$\frac{{k_{nf} }}{{k_{f} }} = \frac{{\overset{\lower0.5em\hbox{$\smash{\scriptscriptstyle\smile}$}}{k}_{{Al_{2} O_{3} ,p1}} + 2\overset{\lower0.5em\hbox{$\smash{\scriptscriptstyle\smile}$}}{k}_{{H_{2} O}} - 2\phi_{{Al_{2} O_{3,p1} }} \left( {\overset{\lower0.5em\hbox{$\smash{\scriptscriptstyle\smile}$}}{k}_{{H_{2} O}} - \overset{\lower0.5em\hbox{$\smash{\scriptscriptstyle\smile}$}}{k}_{{Al_{2} O_{3} ,p1}} } \right)}}{{\overset{\lower0.5em\hbox{$\smash{\scriptscriptstyle\smile}$}}{k}_{{Al_{2} O_{3,p1} }} + 2\overset{\lower0.5em\hbox{$\smash{\scriptscriptstyle\smile}$}}{k}_{{H_{2} O}} + \phi_{{Al_{2} O_{3,p1} }} \left( {\overset{\lower0.5em\hbox{$\smash{\scriptscriptstyle\smile}$}}{k}_{{H_{2} O}} - \overset{\lower0.5em\hbox{$\smash{\scriptscriptstyle\smile}$}}{k}_{{Al_{2} O_{3,p1} }} } \right)}}$$			
Electrical conductivity	$$\frac{{\overset{\lower0.5em\hbox{$\smash{\scriptscriptstyle\smile}$}}{\sigma }_{{\left( {Al_{2} O_{3} - CuO - Cu} \right)H_{2} O}} }}{{\overset{\lower0.5em\hbox{$\smash{\scriptscriptstyle\smile}$}}{\sigma }_{{\left( {Al_{2} O_{3} - CuO} \right)H_{2} O}} }} = \frac{{\overset{\lower0.5em\hbox{$\smash{\scriptscriptstyle\smile}$}}{\sigma }_{Cu,p3} + 2\overset{\lower0.5em\hbox{$\smash{\scriptscriptstyle\smile}$}}{\sigma }_{{\left( {Al_{2} O_{3} - CuO} \right)w}} - 2\phi_{Cu,p3} \left( {\overset{\lower0.5em\hbox{$\smash{\scriptscriptstyle\smile}$}}{\sigma }_{{\left( {Al_{2} O_{3} - CuO} \right)w}} - \overset{\lower0.5em\hbox{$\smash{\scriptscriptstyle\smile}$}}{\sigma }_{Cu,p3} } \right)}}{{\overset{\lower0.5em\hbox{$\smash{\scriptscriptstyle\smile}$}}{\sigma }_{Cu,p3} + 2\overset{\lower0.5em\hbox{$\smash{\scriptscriptstyle\smile}$}}{\sigma }_{{\left( {Al_{2} O_{3} - CuO} \right)w}} + \phi_{Cu,p3} \left( {\overset{\lower0.5em\hbox{$\smash{\scriptscriptstyle\smile}$}}{\sigma }_{{\left( {Al_{2} O_{3} - CuO} \right)w}} - \overset{\lower0.5em\hbox{$\smash{\scriptscriptstyle\smile}$}}{\sigma }_{Cu,p3} } \right)}}$$ where $$\frac{{\overset{\lower0.5em\hbox{$\smash{\scriptscriptstyle\smile}$}}{\sigma }_{{\left( {Al_{2} O_{3} - CuO} \right)w}} }}{{\overset{\lower0.5em\hbox{$\smash{\scriptscriptstyle\smile}$}}{\sigma }_{nf} }} = \frac{{\overset{\lower0.5em\hbox{$\smash{\scriptscriptstyle\smile}$}}{\sigma }_{CuO,p2} + 2\overset{\lower0.5em\hbox{$\smash{\scriptscriptstyle\smile}$}}{\sigma }_{nf} - 2\phi_{CuO,p2} \left( {\overset{\lower0.5em\hbox{$\smash{\scriptscriptstyle\smile}$}}{\sigma }_{nf} - \overset{\lower0.5em\hbox{$\smash{\scriptscriptstyle\smile}$}}{\sigma }_{CuO,p2} } \right)}}{{\overset{\lower0.5em\hbox{$\smash{\scriptscriptstyle\smile}$}}{\sigma }_{CuO,p2} + 2\overset{\lower0.5em\hbox{$\smash{\scriptscriptstyle\smile}$}}{\sigma }_{nf} + \phi_{CuO,p2} \left( {\overset{\lower0.5em\hbox{$\smash{\scriptscriptstyle\smile}$}}{\sigma }_{nf} - \overset{\lower0.5em\hbox{$\smash{\scriptscriptstyle\smile}$}}{\sigma }_{CuO,p2} } \right)}}$$ $$\frac{{\overset{\lower0.5em\hbox{$\smash{\scriptscriptstyle\smile}$}}{\sigma }_{nf} }}{{\overset{\lower0.5em\hbox{$\smash{\scriptscriptstyle\smile}$}}{\sigma }_{{H_{2} O}} }} = \frac{{\overset{\lower0.5em\hbox{$\smash{\scriptscriptstyle\smile}$}}{\sigma }_{{Al_{2} O_{3} }} ,p1 + 2\overset{\lower0.5em\hbox{$\smash{\scriptscriptstyle\smile}$}}{\sigma }_{{H_{2} O}} - 2\phi_{{Al_{2} O_{3} ,p1}} \left( {\overset{\lower0.5em\hbox{$\smash{\scriptscriptstyle\smile}$}}{\sigma }_{{H_{2} O}} - \overset{\lower0.5em\hbox{$\smash{\scriptscriptstyle\smile}$}}{\sigma }_{{Al_{2} O_{3} }} ,p1} \right)}}{{\overset{\lower0.5em\hbox{$\smash{\scriptscriptstyle\smile}$}}{\sigma }_{{Al_{2} O_{3} ,p1}} + 2\overset{\lower0.5em\hbox{$\smash{\scriptscriptstyle\smile}$}}{\sigma }_{{H_{2} O}} + \phi_{{Al_{2} O_{3} ,p1}} \left( {\overset{\lower0.5em\hbox{$\smash{\scriptscriptstyle\smile}$}}{\sigma }_{{H_{2} O}} - \overset{\lower0.5em\hbox{$\smash{\scriptscriptstyle\smile}$}}{\sigma }_{{Al_{2} O_{3} ,p1}} } \right)}}$$			
Basic components	$$\hat{\rho }\;\left( {{\text{kg/m}}^{3} } \right)$$	$$\hat{c}_{p} \;\left( {{\text{J/Kg}}\;{\text{K}}} \right)$$	$$\hat{k}\;\left( {\text{W/mk}} \right)$$	$$\overset{\lower0.5em\hbox{$\smash{\scriptscriptstyle\smile}$}}{\sigma } \;\left( {\Omega {\text{m}}} \right)^{ - 1}$$
CuO	6500	540	18	$6.9\times {10}^{-2}$
H_2_O	997.1	$4180$	$0.6071$	$5.5\times {10}^{-6}$
Cu	8933	385	400	$59.6\times {10}^{6}$
Al_2_O_3_	$3970$	$765$	$40$	$35\times {10}^{6}$

**Figure 3:**
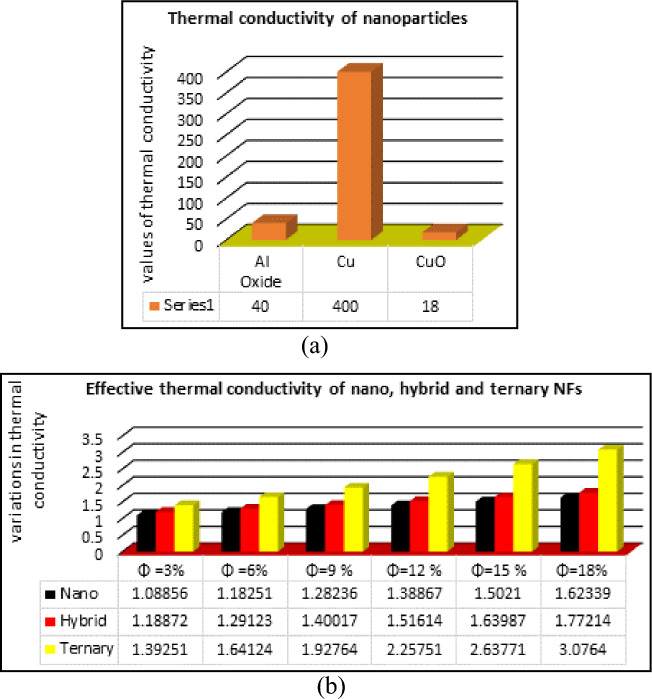
Enhanced thermal conductivity of nano, hybrid and ternary NFs.

Figure 3 is the graphical depiction of the nanoparticles individual thermal conductivity (Fig. 3a) and their impact on thermal conductivity of nano, hybrid and ternary NFs (Fig. 3b). The analysis of the results reveals that thermal conductivity of ternary nanofluid is superior over the previous classes (nano and hybrid nanofluids). For this reason, TNF preferred in the present analysis to investigate their role in the fins efficiency.

### Annular fin energy model

Finally, the below fin energy model obtained after using the above information along with ternary nanofluid characteristics:

9 $$\beta^{\prime \prime } - \alpha_{1} \left( { - \beta + \alpha_{2} } \right)\beta^{{^{\prime \prime } }} + \frac{{k_{f} }}{{k_{ternary} }}\left[ {\begin{array}{*{20}c} {\frac{{\alpha_{1} }}{{\left( {1 + \eta } \right)}}\left( {\beta - \alpha_{2} } \right)\beta^{\prime } + \frac{1}{{\left( {1 + \eta } \right)}}\beta^{\prime } } \\ {\alpha_{1} \beta^{\prime 2} - \frac{{N_{c1} \left( {\beta - \alpha_{2} } \right)^{n + 1} }}{{\left( {1 - \alpha_{2} } \right)^{n} }} - R_{d} \left( {\beta^{4} - \alpha_{2}^{4} } \right)} \\ { - Q_{1} \left( {1 + \gamma_{1} \left( { - \beta + \alpha_{2} } \right)} \right)} \\ { + \frac{{M_{1} \sigma_{ternary} }}{{\sigma_{f} }}\left( {\frac{{ - \beta + \alpha_{2} }}{{ - 1 + \alpha_{2} }}} \right)} \\ \end{array} } \right] = 0$$

$$\frac{{k}_{ternary}}{{k}_{f}}=\left[\begin{array}{c}\frac{\overset{\lower0.5em\hbox{$\smash{\scriptscriptstyle\smile}$}}{k}_{Cu,p3}+2\overset{\lower0.5em\hbox{$\smash{\scriptscriptstyle\smile}$}}{k}_{\left(A{l}_{2}{O}_{3}-CuO\right)w}-2{\phi }_{Cu,p3}\left(\overset{\lower0.5em\hbox{$\smash{\scriptscriptstyle\smile}$}}{k}_{\left(A{l}_{2}{O}_{3}-CuO\right)w}-\overset{\lower0.5em\hbox{$\smash{\scriptscriptstyle\smile}$}}{k}_{Cu,p3}\right)}{\overset{\lower0.5em\hbox{$\smash{\scriptscriptstyle\smile}$}}{k}_{Cu,p3}+2\overset{\lower0.5em\hbox{$\smash{\scriptscriptstyle\smile}$}}{k}_{\left(A{l}_{2}{O}_{3}-CuO\right)w}+{\phi }_{Cu,p3}(\overset{\lower0.5em\hbox{$\smash{\scriptscriptstyle\smile}$}}{k}_{\left(A{l}_{2}{O}_{3}-CuO\right)w}-\overset{\lower0.5em\hbox{$\smash{\scriptscriptstyle\smile}$}}{k}_{Cu,p3})}*\\ \frac{\overset{\lower0.5em\hbox{$\smash{\scriptscriptstyle\smile}$}}{k}_{CuO,p2}+2\overset{\lower0.5em\hbox{$\smash{\scriptscriptstyle\smile}$}}{k}_{nf}-2{\phi }_{CuO,p2}\left(\overset{\lower0.5em\hbox{$\smash{\scriptscriptstyle\smile}$}}{k}_{nf}-\overset{\lower0.5em\hbox{$\smash{\scriptscriptstyle\smile}$}}{k}_{CuO,p2}\right)}{\overset{\lower0.5em\hbox{$\smash{\scriptscriptstyle\smile}$}}{k}_{CuO,p2}+2\overset{\lower0.5em\hbox{$\smash{\scriptscriptstyle\smile}$}}{k}_{nf}+{\phi }_{CuO,p2}(\overset{\lower0.5em\hbox{$\smash{\scriptscriptstyle\smile}$}}{k}_{nf}-\overset{\lower0.5em\hbox{$\smash{\scriptscriptstyle\smile}$}}{k}_{CuO,p2})}*\\ \frac{\overset{\lower0.5em\hbox{$\smash{\scriptscriptstyle\smile}$}}{k}_{A{l}_{2}{O}_{3}},p1+2\overset{\lower0.5em\hbox{$\smash{\scriptscriptstyle\smile}$}}{k}_{{H}_{2}O}-2{\phi }_{A{l}_{2}{O}_{3},p1}\left(\overset{\lower0.5em\hbox{$\smash{\scriptscriptstyle\smile}$}}{k}_{{H}_{2}O}-\overset{\lower0.5em\hbox{$\smash{\scriptscriptstyle\smile}$}}{k}_{A{l}_{2}{O}_{3},p1}\right)}{\overset{\lower0.5em\hbox{$\smash{\scriptscriptstyle\smile}$}}{k}_{A{l}_{2}{O}_{3},p1}+2\overset{\lower0.5em\hbox{$\smash{\scriptscriptstyle\smile}$}}{k}_{{H}_{2}O}+{\phi }_{A{l}_{2}{O}_{3},p1}(\overset{\lower0.5em\hbox{$\smash{\scriptscriptstyle\smile}$}}{k}_{{H}_{2}O}-\overset{\lower0.5em\hbox{$\smash{\scriptscriptstyle\smile}$}}{k}_{A{l}_{2}{O}_{3},p1})}\end{array}\right]$$

$$\frac{{\sigma }_{ternary}}{{\sigma }_{f}}=\left[\begin{array}{c}\frac{\overset{\lower0.5em\hbox{$\smash{\scriptscriptstyle\smile}$}}{\sigma }_{Cu,p3}+2\overset{\lower0.5em\hbox{$\smash{\scriptscriptstyle\smile}$}}{\sigma }_{\left(A{l}_{2}{O}_{3}-CuO\right)w}-2{\phi }_{Cu,p3}\left(\overset{\lower0.5em\hbox{$\smash{\scriptscriptstyle\smile}$}}{\sigma }_{\left(A{l}_{2}{O}_{3}-CuO\right)w}-\overset{\lower0.5em\hbox{$\smash{\scriptscriptstyle\smile}$}}{\sigma }_{Cu,p3}\right)}{\overset{\lower0.5em\hbox{$\smash{\scriptscriptstyle\smile}$}}{\sigma }_{Cu,p3}+2\overset{\lower0.5em\hbox{$\smash{\scriptscriptstyle\smile}$}}{\sigma }_{\left(A{l}_{2}{O}_{3}-CuO\right)w}+{\phi }_{Cu,p3}(\overset{\lower0.5em\hbox{$\smash{\scriptscriptstyle\smile}$}}{\sigma }_{\left(A{l}_{2}{O}_{3}-CuO\right)w}-\overset{\lower0.5em\hbox{$\smash{\scriptscriptstyle\smile}$}}{\sigma }_{Cu,p3})}*\\ \frac{\overset{\lower0.5em\hbox{$\smash{\scriptscriptstyle\smile}$}}{\sigma }_{CuO,p2}+2\overset{\lower0.5em\hbox{$\smash{\scriptscriptstyle\smile}$}}{\sigma }_{nf}-2{\phi }_{CuO,p2}\left(\overset{\lower0.5em\hbox{$\smash{\scriptscriptstyle\smile}$}}{\sigma }_{nf}-\overset{\lower0.5em\hbox{$\smash{\scriptscriptstyle\smile}$}}{\sigma }_{CuO,p2}\right)}{\overset{\lower0.5em\hbox{$\smash{\scriptscriptstyle\smile}$}}{\sigma }_{CuO,p2}+2\overset{\lower0.5em\hbox{$\smash{\scriptscriptstyle\smile}$}}{\sigma }_{nf}+{\phi }_{CuO,p2}(\overset{\lower0.5em\hbox{$\smash{\scriptscriptstyle\smile}$}}{\sigma }_{nf}-\overset{\lower0.5em\hbox{$\smash{\scriptscriptstyle\smile}$}}{\sigma }_{CuO,p2})}*\\ \frac{\overset{\lower0.5em\hbox{$\smash{\scriptscriptstyle\smile}$}}{\sigma }_{A{l}_{2}{O}_{3}},p1+2\overset{\lower0.5em\hbox{$\smash{\scriptscriptstyle\smile}$}}{\sigma }_{{H}_{2}O}-2{\phi }_{A{l}_{2}{O}_{3},p1}\left(\overset{\lower0.5em\hbox{$\smash{\scriptscriptstyle\smile}$}}{\sigma }_{{H}_{2}O}-\overset{\lower0.5em\hbox{$\smash{\scriptscriptstyle\smile}$}}{\sigma }_{A{l}_{2}{O}_{3},p1}\right)}{\overset{\lower0.5em\hbox{$\smash{\scriptscriptstyle\smile}$}}{\sigma }_{A{l}_{2}{O}_{3},p1}+2\overset{\lower0.5em\hbox{$\smash{\scriptscriptstyle\smile}$}}{\sigma }_{{H}_{2}O}+{\phi }_{A{l}_{2}{O}_{3},p1}(\overset{\lower0.5em\hbox{$\smash{\scriptscriptstyle\smile}$}}{\sigma }_{{H}_{2}O}-\overset{\lower0.5em\hbox{$\smash{\scriptscriptstyle\smile}$}}{\sigma }_{A{l}_{2}{O}_{3},p1})}\end{array}\right]$$

The conditions subject to the fin energy model are $\beta =1 and {\beta }^{^{\prime}}=0$, respectively.

## Mathematical analysis of energy model

The final energy model integrated by ternary nanoliquid characteristics, focused magnetic field, heating source, thermal radiation and coefficient of thermal conductivity contains nonlinearities. Although, the model is of second order and first degree but it contains number of nonlinearities $\beta^{4}$ and $\beta^{\prime 2}$ etc. To tackle such terms during the solution, implementation of numerical method is a safe way. Thus, the RKF-45 (see Refs^37,38^.) is adopted for the analysis along with the ingrained physical constraints. The RKF-45 mainly applicable on reduced IVPs^39^ containing linear or nonlinear terms. The main advantage of this scheme is that it is easy to implement to acquire the results with high accuracy with less computational cost, stable and is self-starting scheme. Further, like multi step schemes, this technique do not require to compute first few terms by using some single step methods. The main disadvantages of this technique is that it is not suitable for the stiff system. In comparison with analytical schemes like HAM, the RKF-45^40^ is better suited for such nanofluid models to investigate the results with desirable accuracy.

**Figure 4:**
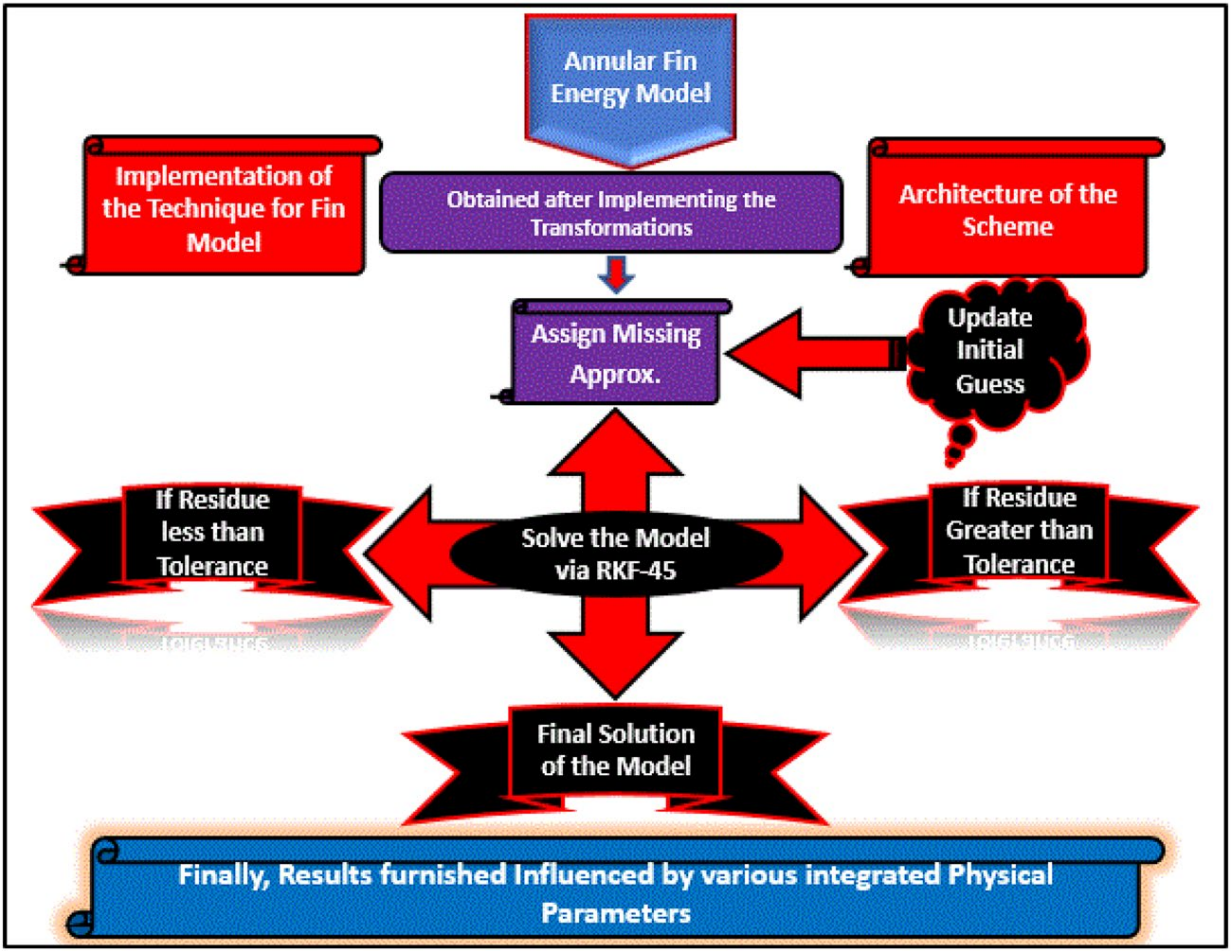
Implementation of RKF-45 for annular fin energy model.

In order to achieve the model output, the complete road map of this technique is demonstrated in Fig. 4.

## Results and discussion

This important section devoted to analyze thermal management in annular fin influenced by the concerned parameters ${\alpha }_{1}, {\alpha }_{2}$, ${N}_{c1}, {M}_{1},{R}_{d}$ and ${\gamma }_{1}$. To predict impactful heat transfer trends, the results furnished for common, fluid, mono nano, hybrid with two types of nanoparticles and ternary nanofluids.

### Thermal management in annular fin under physical constraints

**Figure 5:**
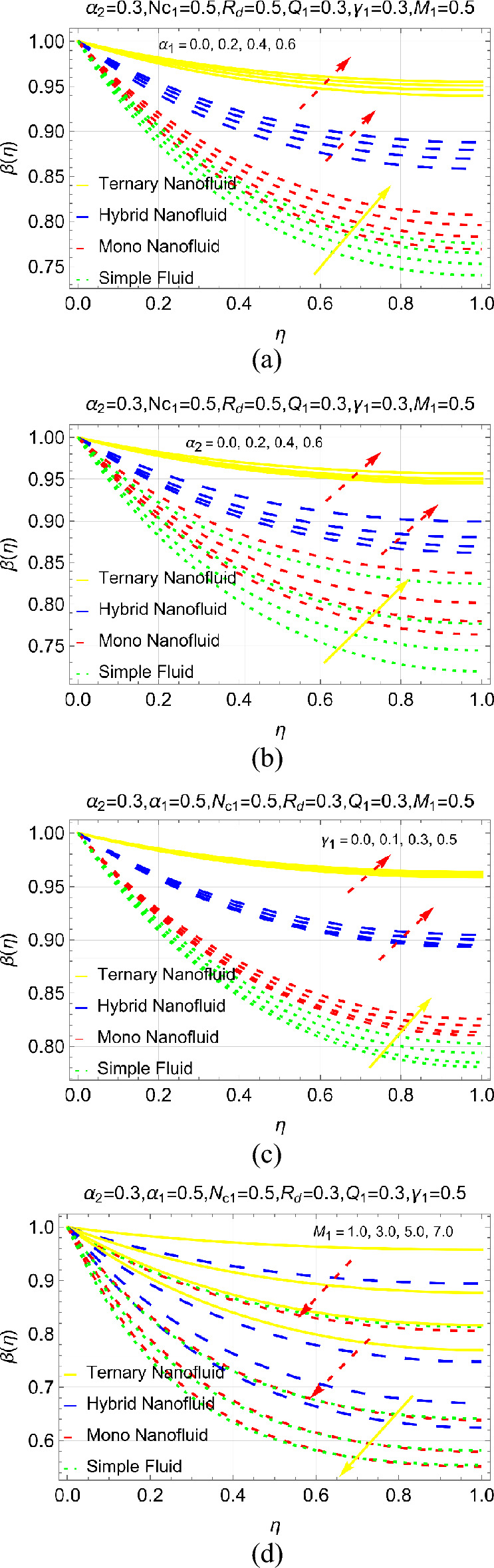
Thermal management due to (**a**) ${\alpha }_{1}$ (**b**) ${\alpha }_{2}$ (**c**) ${\gamma }_{1}$ and (**d**) ${M}_{1}$.

Thermal management due to increasing ${\alpha }_{1}$ (coefficient of thermal conductivity), ${\alpha }_{2}$ (designates the ambient temperature), ${\gamma }_{1}$ and ${M}_{1}$ (directed magnetic forces). The temperature trends under varying coefficient of thermal conductivity (${\alpha }_{1}$) under various stages demonstrated in Fig. 5a. The results demonstrating that the heat transmission of fin enhances as the values of ${\alpha }_{1}$ increases. Physically, increment in coefficient of thermal conductivity enhances thermal conductivity of fin. As a consequence, internal energy of the material boosts which ultimately increase the heat transmission rate of fin. It is observed that the heat transfer process at the surface if very slow. It is worth to mention that, in this case the heat transfers due to two modes known as conduction and convection. Physically, slow heat transfer occurs in the case of conduction because the particles gain heat and then transfer to the neighboring particles as a results the fin efficiency rises slowly. After that, the convection becomes dominant and the fin temperature rises rapidly. Convection is a fast heat transmission mechanism thus; due to high convection the temperature is observed optimum towards the outer position of the fin i.e. $\eta =1$.

The results against ${\alpha }_{2}, {\gamma }_{1}$ and ${M}_{1}$ organized in Fig. 5b–d, respectively. It is examined that the fin efficiency can be improved by enlarging the ambient temperature number ${\alpha }_{2}$. It is boosted towards the fin outer position for greater ${\alpha }_{2}$ and ${\gamma }_{1}$. Moreover, the heat efficiency in ternary nanofluid is dominant than hybrid, nano and conventional fluid, respectively. This shows that use of ternary nanoliquid is better way to enhance the fin efficiency. Figure 5d describes that the fin temperature can be controlled by using stronger magnetic forces. The efficiency drops very rapidly for simple fluid and a very slow decrement observed for ternary nanoliquid followed by hybrid and mono nanoliquids, respectively.

**Figure 6:**
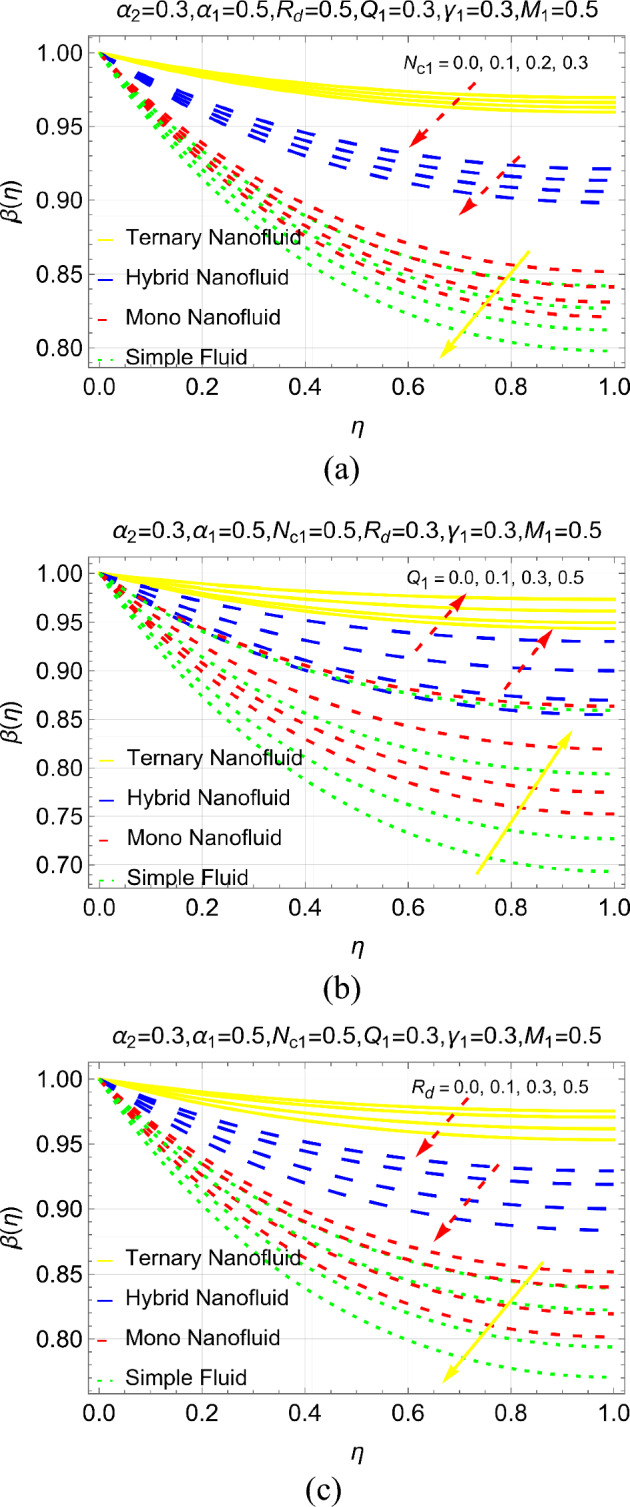
Thermal management under (**a**) ${N}_{c1}$ (**b**) ${Q}_{1}$ and (**c**) ${R}_{d}$.

Figure 6a–c portrayed to investigate the heat efficiency under radiative conductive number (${N}_{c1}$), heating source (${Q}_{1}$) and radiation number (${R}_{d}$). It is investigated that thermal efficiency of fin can be achieved up to the mark by strengthening internal energy of the fin. Physically, increase in internal energy of fin provide extra energy to the fin’s particles which transfer it to the surrounding particles and the fin efficiency improved. Moreover, it can be seen that high efficacy of the fin is subject to ternary nanoliquid. Further, radio-conductive and radiation numbers are observed as source of fin cooling in the particular analysis. The optimum fin cooling can be achieved by strengthening ${N}_{c1}$ and ${R}_{d}$. For the fin cooling purpose, simple fluid is better however; nano, hybrid and ternary nanoliquids lead to slow declines in the heat transmission.

**Figure 7:**
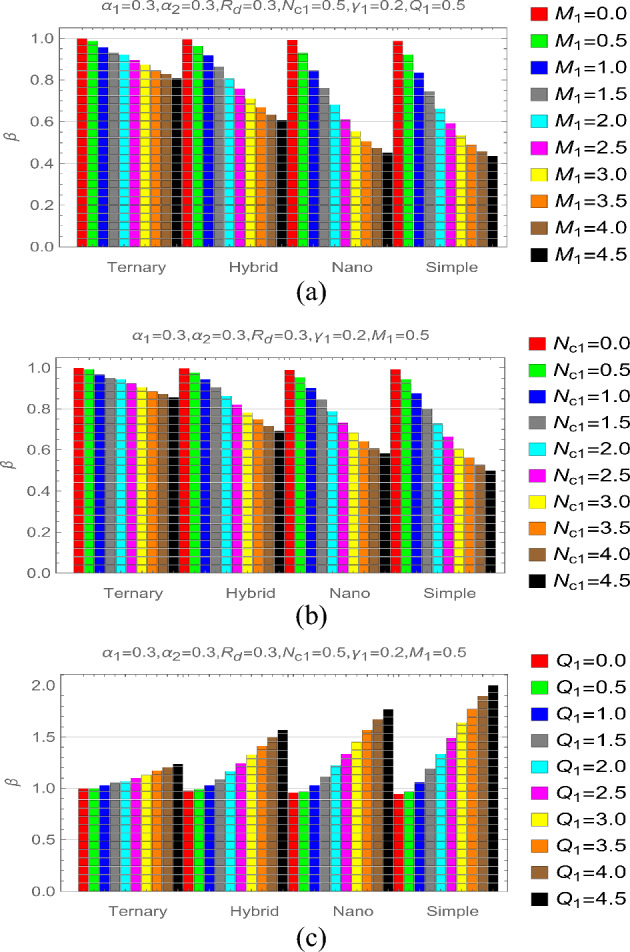
Thermal stress via (**a**) ${M}_{1}$ (**b**) ${N}_{c1}$ and (**c**) ${Q}_{1}$.

Figure 7a–c elucidating the impact of ${M}_{1}, {N}_{c1}$ and ${Q}_{1}$ for different parametric stages. The results from Fig. 7a,b against stronger ${M}_{1}$ and ${N}_{c1}$ showing that the heat performance of fin reduced against stronger directed magnetic forces and hence it is a good way to control the heat transfer rate of fin. The maximum decrement is noticed for simple type of fluid under both varying ${M}_{1}$ and ${N}_{c1}$. On the other way, the heating source ${Q}_{1}$ is a key for thermal enhancement and playing the role of catalytic tool to boost the fin energy performance. These results demonstrated in Fig. 7c for feasible parametric stages. Physically, internal source produces heat due to which the conductive process becomes fast and transmits the heat to the neighboring particles rapidly.

**Figure 8:**
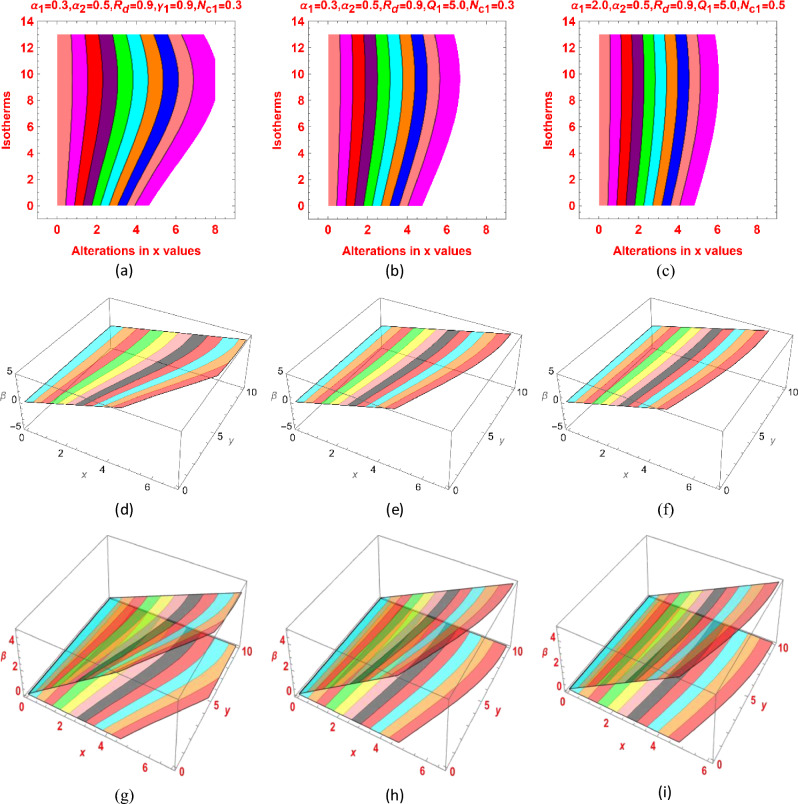
Isotherms contour for (**a**,**d**,**g**) ${R}_{d}=0.9$ (**b**,**e**,**h**) ${Q}_{1}=5.0$ and (**c**,**f**,**i**) ${\alpha }_{1}=2.0$

Figure 8a–i decorating the isotherm contours for different physical effects. The isotherms are more curved for ${R}_{d}=0.9$ however; these trends become flatten as the value of ${Q}_{1}$ and ${\alpha }_{1}$ grows up. Other parameters kept fixed as mentioned in the graphical results.

### Study and code validation

To ensure the validity of study and model, the current model results compared with the reported data (Table 4). The obtained results from the present model are valid and aligned with data of Arslanturk^23^ under convective fin condition. To make the model compatible with Arslanturk^23^, some parametric restrictions imposed on the model and these values are ${\alpha }_{1}=0.3, {N}_{c1}=0.6, {R}_{d}=0, {Q}_{1}=0, {\gamma }_{1}=0, {\alpha }_{2}=0$ and ${M}_{1}=0$. The data computed at various stages of fin surface ($\eta =0.0$ to $\eta =0.3$) and executed results provided a fine validation.

**Table 4:** The validation of the model with previously published data of Arslanturk^23^.

$\eta$	Present results	Arslanturk^7^
$0.0$	$1.00000$	$1.0000$
$0.1$	$0.947672$	$0.9477$
$0.2$	$0.903621$	$0.9036$
$0.3$	$0.866825$	$0.8668$

## Conclusions

This research deals with the simulation of annular fin heat performance with physical influence of coefficient of thermal conductivity, natural convection, magnetic forces and thermal radiations effects. The conventional model updated via modified Tiwari–Das model and then the results simulated using various parametric stages. The core output of the study is as under:

- The fin performance can be improved by enhancing coefficient of thermal conductivity ${\alpha }_{1}, {\alpha }_{2}$ and ${\gamma }_{1}$ and high fin efficiency observed for ternary nanofluid.
- The magnetic forces and thermal radiations effects increase the cooling performance of annular fin.
- Internal heating source ${Q}_{1}$ is a key to enhance the heat transmission efficacy of the fin with suitable ranges.
- Ternary nanofluid greatly influenced in the fin performance throughout the study with different parameters.
- The study and code validated with existing data and achieved high degree validity under compatible results.

## Data Availability

The datasets used and/or analysed during the current study available from the corresponding author on reasonable request.

## References

[1] Sadri S, Raveshi MR, Amiri S (2012). Efficiency analysis of straight fin with variable heat transfer coefficient and thermal conductivity. J. Mech. Sci. Technol..

[2] Srinivasa RG, Vathsav KS, Koushik R (2022). Numerical analysis of convective heat transfer on straight finned tube with different nano fluids. Materialstoday.

[3] Din ZU, Ali A, Khan ZA, Zaman G (2022). Heat transfer analysis: Convective-radiative moving exponential porous fins with internal heat generation. Math. Biosci. Eng..

[4] Gireesha BJ, Sowmya G, Khan MI, Öztop HF (2020). Flow of hybrid nanofluid across a permeable longitudinal moving fin along with thermal radiation and natural convection. Comput. Methods Prog. Biomed..

[5] Arshad A, Ali HM, Ali M, Manzoor S (2017). Thermal performance of phase change material (PCM) based pin-finned heat sinks for electronics devices: Effect of pin thickness and PCM volume fraction. Appl. Therm. Eng..

[6] Ali HM, Jabbal M, Verdin PG (2018). Thermal management of electronics devices with PCMs filled pin-fin heat sinks: A comparison. Int. J. Heat Mass Transf..

[7] Ullah I, Shukat S, Albakri A, Khan H, Galal AM, Jamshed W (2023). Thermal performance of aqueous alumina–titania hybrid nanomaterials dispersed in rotating channel. Int. J. Mod. Phys. B.

[8] Murtaza R, Hussain I, Rehman Z, Khan I, Andualem M (2022). Thermal enhancement in Falkner-Skan flow of the nanofluid by considering molecular diameter and freezing temperature. Sci. Rep..

[9] Ikram U (2022). Heat transfer enhancement in Marangoni convection and nonlinear radiative flow of gasoline oil conveying Boehmite alumina and aluminum alloy nanoparticles. Int. Commun. Heat Mass Transf..

[10] Ullah I (2022). Activation energy with exothermic/endothermic reaction and Coriolis force effects on magnetized nanomaterials flow through Darcy–Forchheimer porous space with variable features. Waves Random Complex Media.

[11] Gul T, Bilal M, Alghamdi W, Asjad MI, Abdeljawad T (2021). Hybrid nanofluid flow within the conical gap between the cone and the surface of a rotating disk. Sci. Rep..

[12] Rahman MM, Chamkha AJ, Elmasry Y, Pasha AA, Sadeghi MS (2022). The heat transfer behavior of MHD micro-polar MWCNT-Fe3O4/water hybrid nano-fluid in an inclined. Case Stud. Therm. Eng..

[13] Haq I, Muhammad B, Ahammad NA, Ghoneim ME, Ali A (2022). Mixed convection nanofluid flow with heat source and chemical reaction over an inclined irregular surface. ACS Omega.

[14] Jan RU, Khan H, Alam MM (2022). Improving the thermal performance of (ZnO-Ni/H2O) hybrid nanofluid flow over a rotating system: The applications of Darcy Forchheimer theory. Waves Random Complex Media.

[15] Pattanaik PC, Mishra S, Jena S, Pattnaik PK (2022). Impact of radiative and dissipative heat on the Williamson nanofluid flow within a parallel channel due to thermal buoyancy. Part N J. Nanomater. Nanoeng. Nanosyst..

[16] Tinker S, Sharma RP (2022). Simulation of time-dependent radiative heat motion over a stretching/shrinking sheet of hybrid nanofluid: Stability analysis for dual solutions. Part N J. Nanomater. Nanoeng. Nanosyst..

[17] Hayat T, Alsaedi A (2022). Optimization of entropy production in flow of hybrid nanomaterials through Darcy–Forchheimer porous space. J. Therm. Anal. Calorim..

[18] Mishra SR, Bég OA, Khan UF, Umavathi JC (2022). Axisymmetric radiative titanium dioxide magnetic nanofluid flow on a stretching cylinder with homogeneous/heterogeneous reactions in Darcy–Forchheimer porous media: Intelligent nanocoating simulation. Mater. Sci. Eng. B.

[19] Ikram U, Refat U, Alqarni SM, Xia WF, Taseer M (2022). Combined heat source and zero mass flux features on magnetized nanofluid flow by radial disk with the applications of Coriolis force and activation energy. Int. Commun. Heat Mass Transf..

[20] Pattnaika KP, Pattnaika RJ, Mishra SR (2022). Illustration of low-pressure gradient on the MHD flow of viscous fluid over a flat plate: The Homotopy Perturbation Method. Waves Random Complex Media.

[21] Kumar RV, Sowmya G, Essa FA, Prasannakumara BC, Alsehli M, Saleh B (2022). Thermal analysis of an annular fin under multi-boiling heat transfer coefficient using differential transform method with Pade approximant (DTM-Pade). Part E J. Process Mech. Eng..

[22] Mallick, A., Ghosal, S., Sarkar, P. K. & Ranjan, R. Homotopy perturbation method for thermal stresses in an annular fin with variable thermal conductivity. *J. Therm. Stress.* 110–132 (2014).

[23] Arslanturk C (2009). Correlation equations for optimum design of annular fins with temperature dependent thermal conductivity. Heat Mass Transf..

[24] Reddy NK, Sankar M (2022). Buoyant heat transfer of nanofluids in a vertical porous annulus: a comparative study of different models. Int. J. Numer. Methods Heat Fluid Flow.

[25] Senapati JR, Dash SK, Roy S (2016). 3D numerical study of the effect of eccentricity on heat transfercharacteristics over horizontal cylinder fitted with annular fins. Int. J. Therm. Sci..

[26] Torabi M, Yaghoobi H, Colantoni A, Biondi P, Boubaker K (2013). Analysis of radiative radial fin with temperature-dependent thermal conductivity using nonlinear differential transformation methods. Chin. J. Eng..

[27] Rai N, Hegde RN (2020). A modified correlation to predict heat transfer coefficient in a horizontal ‘coil in shell heat exchanger’ with circular fins using nanofluids. AIP Conf. Proc..

[28] Poursharif Z, Salarian H, Javaherdeh K, Nimvari ME (2020). Numerical simulation of heat transfer on nanofluid flow in an annular pipe with simultaneous embedding of porous discs and triangular fins. J. Chin. Inst. Eng..

[29] Haq RU, Shah SS, Algehyne EA, Tlili I (2020). Heat transfer analysis of water based SWCNTs through parallel fins enclosed by square cavity. Int. Commun. Heat Mass Transf..

[30] Hamida MBB, Hatami M (2021). Investigation of heated fins geometries on the heat transfer of a channel filled by hybrid nanofluids under the electric field. Case Stud. Therm. Eng..

[31] Manohar GR, Venkatesh P, Gireesha BJ, Madhukesh JK, Ramesh GK (2021). Dynamics of hybrid nanofluid through a semi spherical porous fin with internal heat generation. Partial Differ. Equ. Appl. Math..

[32] Turkyilmazoglu M (2015). Stretching/shrinking longitudinal fins of rectangular profile and heat transfer. Energy Convers. Manag..

[33] Jalili B, Aghaee N, Jalili P, Ganji DD (2022). Novel usage of the curved rectangular fin on the heat transfer of a double-pipe heat exchanger with a nanofluid. Case Stud. Therm. Eng..

[34] Naphon P, Nakharintr L (2013). Heat transfer of nanofluids in the mini-rectangular fin heat sinks. Int. Commun. Heat Mass Transf..

[35] Ullah I, Ullah S, Ali A, Shah SI, Weera W (2022). Heat transfer analysis from moving convection-radiative triangular porous fin exposed to heat generation. Case Stud. Therm Eng..

[36] Sowmya, G., Gamaoun, F., Abdulrahman, A., Kumar, R. S. V. & Prasannakumara, B. C. Significance of thermal stress in a convective-radiative annular fin with magnetic field and heat generation: application of DTM and MRPSM. *Propuls. Power Res.* 1–17 (2022).

[37] Khalid AMA, Ashraf W, Yassen MF, Jamshed W (2023). Applied heat transfer modeling in conventional hybrid (Al2O3-CuO)/C2H6O2 and modified-hybrid nanofluids (Al2O3-CuO-Fe3O4)/C2H6O2 between slippery channel by using least square method (LSM). AIMS Math..

[38] AlBaidani MM, Mishra NK, Alam MM, Zahrani AAA, Ali A (2023). Numerical analysis of magneto-radiated annular fin natural-convective heat transfer performance using advanced ternary nanofluid considering shape factors with heating source. Case Stud. Therm Eng..

[39] Mashael MA, Kumar NM, Ahmad Z, Haq EU (2023). Numerical study of thermal enhancement in ZnO-SAE50 nanolubricant over a spherical magnetized surface influenced by Newtonian heating and thermal radiation. Case Stud. Therm. Eng..

[40] Adnan. Heat transfer inspection in [(ZnO-MWCNTs)/water-EG(50:50)]hnf with thermal radiation ray and convective condition over a Riga surface. *Waves Random Complex Media* (2022). 10.1080/17455030.2022.2119300

